# G Protein-Coupled Receptor GPR35 Suppresses Lipid Accumulation in Hepatocytes

**DOI:** 10.1021/acsptsci.1c00224

**Published:** 2021-11-30

**Authors:** Li-Chiung Lin, Tezz Quon, Susanna Engberg, Amanda E. Mackenzie, Andrew B. Tobin, Graeme Milligan

**Affiliations:** † The Centre for Translational Pharmacology, Institute of Molecular, Cellular and Systems Biology, College of Medical, Veterinary and Life Sciences, University of Glasgow, Glasgow G12 8QQ, United Kingdom; ‡ Discovery Biology, Discovery Sciences, R&D, AstraZeneca, Pepparedsleden 1, 431 83 Mölndal, Sweden

**Keywords:** G protein-coupled receptor, GPR35, fatty liver disease, hepatocyte, species orthologue

## Abstract

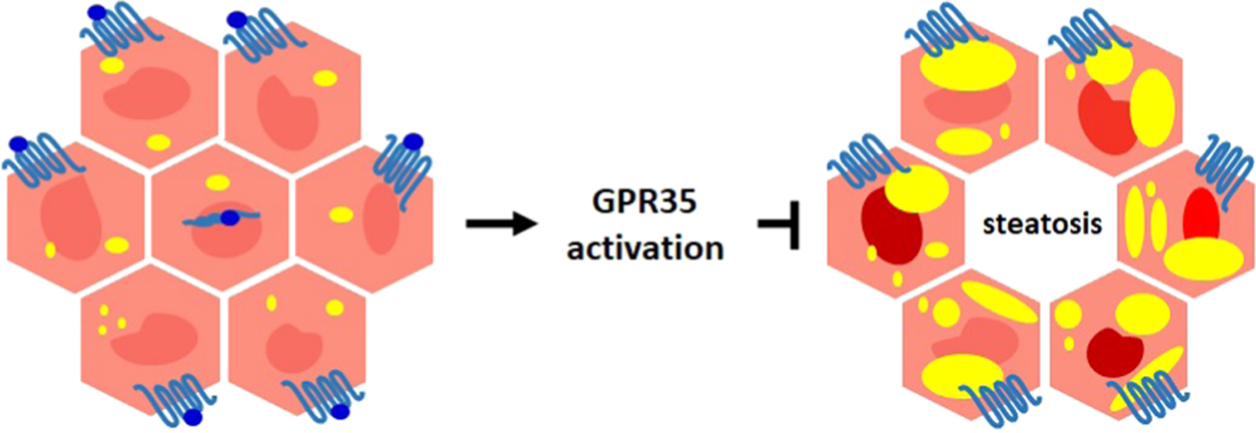

Although prevalent, nonalcoholic fatty liver disease is not currently treated effectively with medicines. Initially, using wild-type and genome-edited clones of the human hepatocyte cell line HepG2, we show that activation of the orphan G protein-coupled receptor GPR35 is both able and sufficient to block liver X-receptor-mediated lipid accumulation. Studies on hepatocytes isolated from both wild-type and GPR35 knock-out mice were consistent with a similar effect of GPR35 agonists in these cells, but because of marked differences in the pharmacology of GPR35 agonists and antagonists at the mouse and human orthologues, as well as elevated basal lipid levels in hepatocytes from the GPR35 knock-out mice, no definitive conclusion could be reached. To overcome this, we generated and characterized a transgenic knock-in mouse line in which the corresponding human GPR35 splice variant replaced the mouse orthologue. In hepatocytes from these humanized GPR35 mice, activation of this receptor was shown conclusively to prevent, and also reverse, lipid accumulation induced by liver X-receptor stimulation. These studies highlight the potential to target GPR35 in the context of fatty liver diseases.

Nonalcoholic fatty liver disease (NAFLD) has increased in prevalence in parallel with the global epidemic of obesity.^1^ NAFLD encompasses a range of conditions from the initial build-up of fat in hepatocytes within the liver (steatosis), to the additional development of inflammation (steatohepatitis) to fibrosis and may potentially result subsequently in liver cirrhosis. While weight loss via diet management represents an effective mitigation strategy, various drug-based interventions, although not currently approved for clinical use, are being explored and these include insulin-sensitizing and other antidiabetic treatments. Given the importance of G protein-coupled receptors (GPCRs) to virtually all aspects of physiological control, the growing understanding of the roles they play in homeostatic control of metabolism,^2,3^ and the success of targeting a wide range of GPCRs in disease settings via small-molecule medicines, it is not surprising that numerous commentators have highlighted potential opportunities in this area for the treatment of NAFLD.^4−9^

GPR35 is an orphan GPCR that can be activated with modest potency by a variety of natural products and endogenous mediators, including kynurenic acid.^10,11^ However, initial reports of the ability of other endogenous molecules, for example, the chemokine CXCLl7,^12^ to selectively activate GPR35 have not been reproduced.^13,14^ A major challenge in efforts to explore roles of GPR35 as a potential therapeutic target is that the pharmacology of both endogenous and synthetic ligands that can either activate or block this receptor is markedly different in rodent preclinical species compared to human^10,11^ and hence great care must be given to appropriately define the contribution of this receptor in cells, cell lines, and tissues from different species. Moreover, unlike mice, which express a single GPR35 isoform, humans express an additional isoform that possesses a 31 amino acid N-terminal extension.^10^ Herein, we explore the role of GPR35 in countering liver X-receptor (LXR)-mediated lipid deposition in both human hepatocyte-like cell lines and primary hepatocytes derived from wild-type and various transgenic mouse lines. To overcome the challenges of species variation in the pharmacology of GPR35, these include a transgenic “knock-in” line in which we replaced mouse GPR35 with the equivalent splice variant of the human orthologue. Using the prisms of both clearly defined pharmacology and genetic engineering and genome-editing approaches, we conclude that activation of GPR35 may be a productive avenue to target NAFLD.

## Results

**Figure 1:**
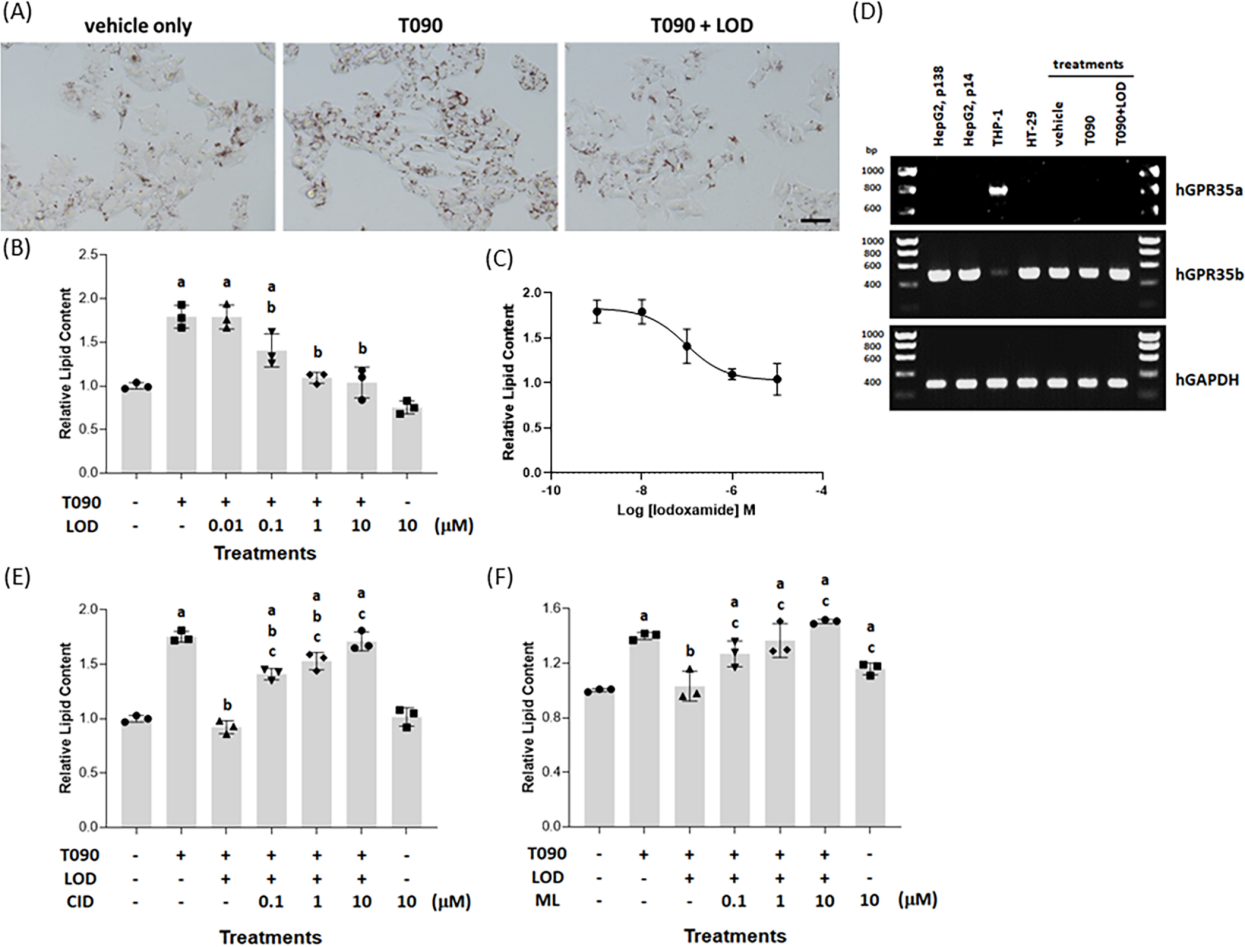
Lodoxamide suppresses LXR-mediated accumulation of lipid in HepG2 cells: This is potentially a GPR35-mediated effect. HepG2 cells (scale bar = 100 μm) were treated for 48 h with the LXR activator T0901317 (4 × 10^–6^ M) or with T0901317 and various concentrations of lodoxamide (1 × 10^–8^–1 × 10^–5^ M). Subsequently, cells were stained with Oil Red O and visualized (lodoxamide = 1 × 10^–5^ M) (A) or lipid-fixed Oil Red O was solubilized and quantified by measuring absorbance at 510 nm (B). The effect of varying concentrations of lodoxamide is shown (C). Data are presented as representative images (A) or mean ± SEM, *n* = 3 (B, C). (D) RT-PCR studies identified expression of the human GPR35b slice variant but not GPR35a by HepG2 cells and HT-29 cells, while THP-1 monocyte-like cells expressed GPR35a. Anticipated base pair (bp) sizes, GPR35a, 737 bp; GPR35b, 464 bp. mRNA levels of GPR35b in HepG2 cells were unaffected by treatment with T0901317 with or without lodoxamide. Human (h)GAPDH provided an internal control. Co-addition of the human GPR35 antagonists CID-2745687 (E) or ML-145 (F) prevented the effect of lodoxamide on lipid accumulation (*p* < 0.05, a: versus vehicle, b: versus T0901317, and c: versus T0901317/lodoxamide).

Treatment of human liver HepG2 cells with the liver X-receptor (LXR) activator *N*-(2,2,2-trifluoroethyl)-*N*-[4-[2,2,2-trifluoro-1-hydroxy-1-(trifluoromethyl)ethyl]phenyl]benzenesulfonamide (T0901317) (Figure S1) (4 × 10^–6^ M, 48 h) resulted in substantially increased (*p* < 0.05) deposition of triglycerides and other lipids, as measured by staining with the diazo dye Oil Red O. This was evident both by direct observation of the cells (Figure 1A) and when assessed quantitatively following extraction of the dye from cell cultures (Figure 1B). Co-incubation of HepG2 cells with T0901317 and various concentrations of 2-[2-chloro-5-cyano-3-(oxaloamino)anilino]-2-oxoacetic acid (lodoxamide) (Figure S1) resulted in a significant (*p* < 0.05) and concentration-dependent reduction in the lipid accumulation induced by T0901317 (Figure 1B). This effect of lodoxamide was achieved with high potency (EC_50_ = 9.5 ± 0.08 × 10^–8^ M) and, at maximally effective concentrations of lodoxamide, the effect of T0901317 was fully suppressed (Figure 1C). By contrast, at the highest concentration employed (1 × 10^–5^ M), without co-addition of T0901317, lodoxamide had no significant effect but tended toward additional reduction of basal lipid accumulation (Figure 1B).

Although frequently described generally as a mast cell stabilizer, an identified molecular target of lodoxamide is the orphan G protein-coupled receptor GPR35^11^ and this ligand displays high potency at each of the human splice variants (short isoform = GPR35a, longer isoform = GPR35b) of this receptor.^15,16^ To assess whether GPR35 might indeed be the relevant molecular target in this observed effect, we initially assessed the expression of GPR35 by HepG2 cells. HT-29 human colon cancer cells are widely used as a cell line expressing GPR35 endogenously.^17−19^ These expressed high levels of mRNA encoding the GPR35b isoform (Figure 1D). HepG2 cells also expressed high levels of mRNA encoding the GPR35b isoform, which was similar throughout passage number (Figure 1D), and levels of this mRNA were essentially unaffected by exposure to T0901317 or T0901317 plus lodoxamide (Figure 1D). We were unable to detect expression of the GPR35a isoform by HepG2 cells, although this splice variant was the predominant form expressed by the human monocytic acute leukemia cell line THP-1^12,13^ (Figure 1D). Chemically distinct ligands, exemplified by 1-(2,4-difluorophenyl)-5-[[2-[[(1,1-dimethylehyl)amino]thioxomethyl]hydrazinylidene]methyl]-1*H*-pyrazole-4-carboxylic acid methyl ester (CID-2745687) (Figure S1) and 2-hydroxy-4-[4-(5*Z*)-5-[(*E*)-2-methyl-3-phenylprop-2-enylidene]-4-oxo-2-sulfanylidene-1,3-thiazolidin-3-yl]butanoylaminobenzoic acid (ML-145) (Figure S1), have been described as antagonists of GPR35.^20−22^ Although neither of these ligands has measurable affinity at the mouse orthologue of GPR35, both have high affinity for the human isoforms of this receptor^21,23^ with *K*_i_ values calculated from competition studies against a [^3^H]radiolabeled agonist of 8.7 × 10^–9^ M (ML-145) and 4.2 × 10^–8^ M (CID-2745687).^24^ We, therefore, next assessed whether CID-2745687 and/or ML-145 could prevent the effect of lodoxamide on LXR-induced lipid accumulation in HepG2 cells. They both did so and in each case in a concentration-dependent fashion (Figure 1E,F).

**Figure 2:**
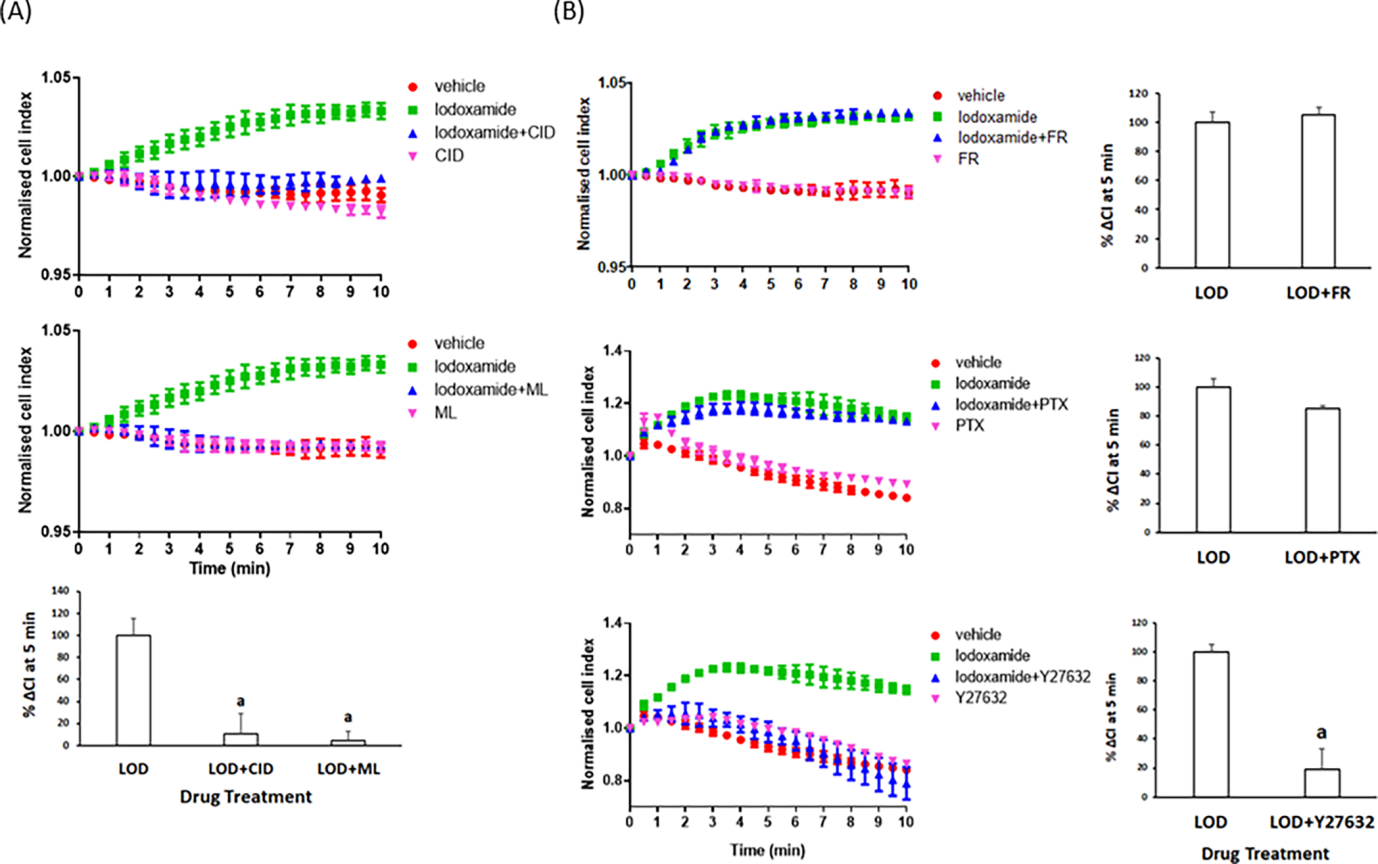
Cellular impedance measurements confirm the function of GPR35 in HepG2 cells. HepG2 cells cultured in xCELLigence microtitre plate wells were able to measure alterations of electrical impedance over time. (A) Once basal conditions were established, vehicle (red), iodoxamide (green) (upper and middle panels), CID-2745687 (pink) (upper panel), or ML-145 (pink) (middle panel) was added either alone or in combination with lodoxamide (blue) (upper and middle panels) and alterations in the signal measured over time. Lower panel: Difference in the “cell index” measured at 5 min treatment with the effect of lodoxamide presented as 100%. (a) *p* < 0.05 versus lodoxamide. (B) Various inhibitors of signaling pathways were added (FR = FR900359, 1 × 10^–7^ M) (upper panel), PTX = Pertussis toxin, 100 ng/mL (middle panel), and Y27632, 1 × 10^–5^ M (lower panel). FR and Y27632 were added 30 min before lodoxamide and PTX 24 h before. Right-hand panels: Difference in the “cell index” measured at 5 min treatment with the effect of lodoxamide presented as 100%. a: *p* < 0.05 versus lodoxamide.

To expand measurements on potential GPR35 activity in HepG2 cells, we turned to a “label-free” assay system. Here, cells grown as a monolayer on a support able to record alterations in electrical conductance (cellular impedance) respond over time as they are challenged with various ligands.^25^ Using an xCELLigence reader in this manner, addition of a low concentration of lodoxamide (1 × 10^–8^ M) produced a time-dependent increase in the signal that reached a plateau within 5 min (Figure 2). This response was absent following co-addition of either CID-2745687 or ML-145 (each at 1 × 10^–5^ M) with lodoxamide (Figure 2A), while neither antagonist generated a response distinct from the vehicle when added alone (Figure 2A). GPR35 signaling mechanisms have been relatively poorly characterized to date.^11,23^ To attempt to define signaling pathways linked to the observed alteration in cellular impedance produced by lodoxamide, we pretreated cells with either the G_q_/G_11_ inhibitor FR900359^26^ or the G_i_-inhibitor Pertussis toxin. Although treatment with FR900359 was without effect on the response to lodoxamide, Pertussis toxin produced a small although a statistically nonsignificant reduction in the response to lodoxamide (Figure 2B). GPR35 is able to effectively activate the G protein Gα_13_.^23^ This is routinely linked to the regulation of Rho-kinases and hence the actin cytoskeleton of cells.^22^ Although there are no direct small-molecule inhibitors of Gα_13_, treatment with the Rho-kinase inhibitor Y27632^27,28^ all but eliminated the response to lodoxamide (Figure 2B).

**Figure 3:**
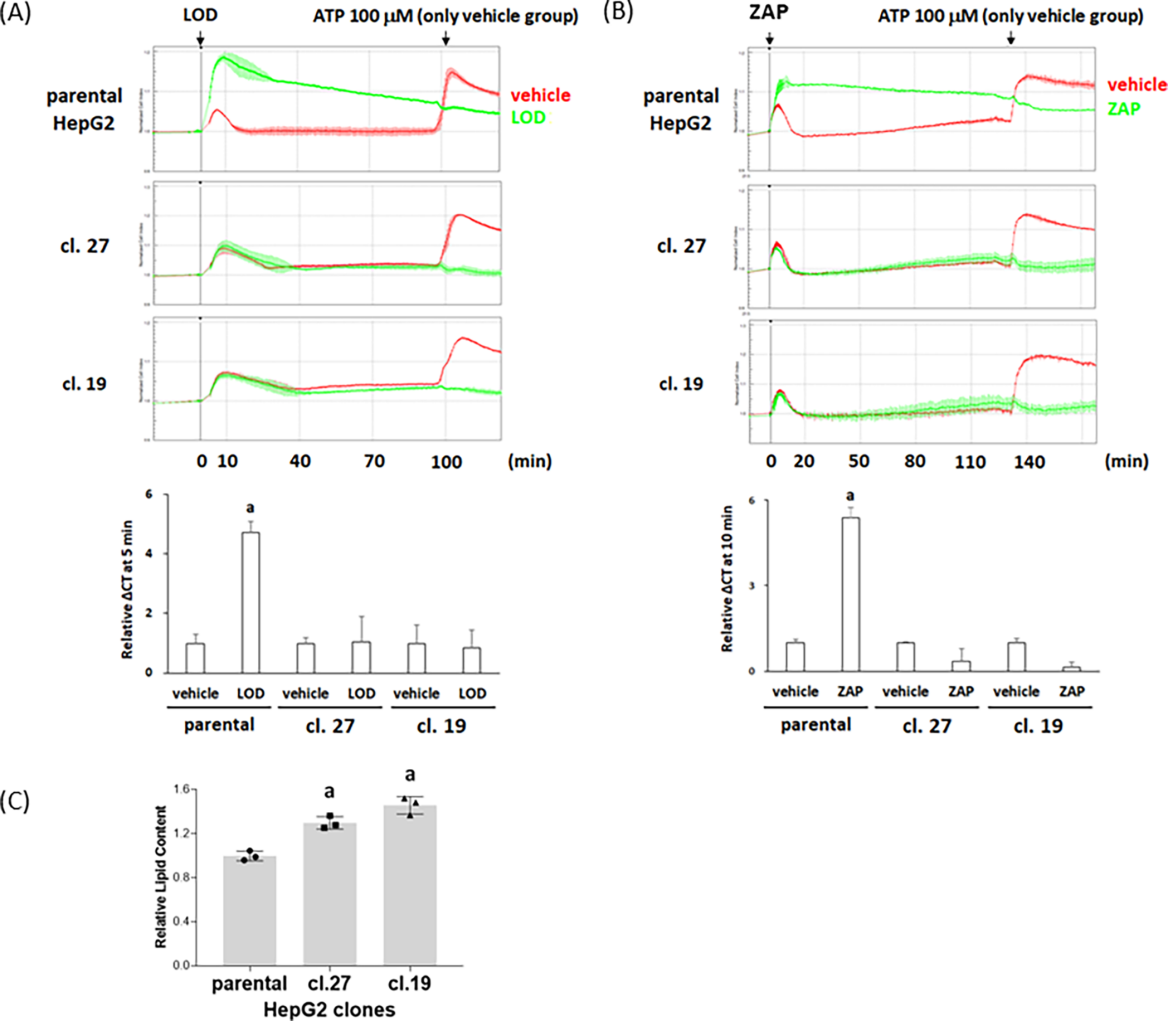
Characterization of GPR35 genome-edited HepG2 clones. Genome-edited clones of HepG2 cells were generated to target the expression of GPR35 (see Figure S2 for details). Parental HepG2 cells and those from clones 19 and 27 were assessed via alterations in electrical impedance, as in Figure 2. Both 1 × 10^–5^ M lodoxamide (A) and zaprinast (B) produced positive signals in parental but not in either clone 19 or 27. By contrast, subsequent addition of ATP showed that cells from each of the lines were capable of generating a clear response. Upper panels: exemplar traces of effect over time. Lower panels: relative effect of lodoxamide and zaprinast measured, as in Figure 2. Data are presented as mean ± range, *n* = 2. (C) Relative basal lipid content of parental HepG2 cells and both clones 19 and 27. a: *p* < 0.05 versus parental.

To confirm that the effects of lodoxamide in such assays truly reflected pharmacological activation of GPR35, we employed CRISPR-Cas9 targeting to generate clones of HepG2 cells lacking expression of full-length GPR35b (Figure S2). In individual clones that sequencing demonstrated to each contain one large deletion and at least one additional smaller deletion that were within the coding exon and resulted in an out-of-frame sequence, a GPR35 PCR fragment only of smaller size compared to wild-type was detected (Figure S2). In such clones (clone 19 and clone 27), alteration in electrical conductance in response to lodoxamide was lacking (Figure 3A). This was also the case when a distinct GPR35 activator zaprinast^23,29^ was employed (Figure 3B). This did not reflect an inability of cells of these clones to respond to a suitable stimulus. In both wild-type HepG2 cells and the GPR35 genome-edited clones, ATP, added to activate P2Y purinoceptors that are expressed by virtually all cell lines in tissue culture, enhanced electrical conductance and the extent and characteristics of response over time to addition of ATP were similar in each line (Figure 3A,B). Interestingly, the basal lipid content of cells of the clones of HepG2 cells lacking GPR35 was significantly (*p* < 0.05) higher than cells of the parental line (Figure 3C).

**Figure 4:**
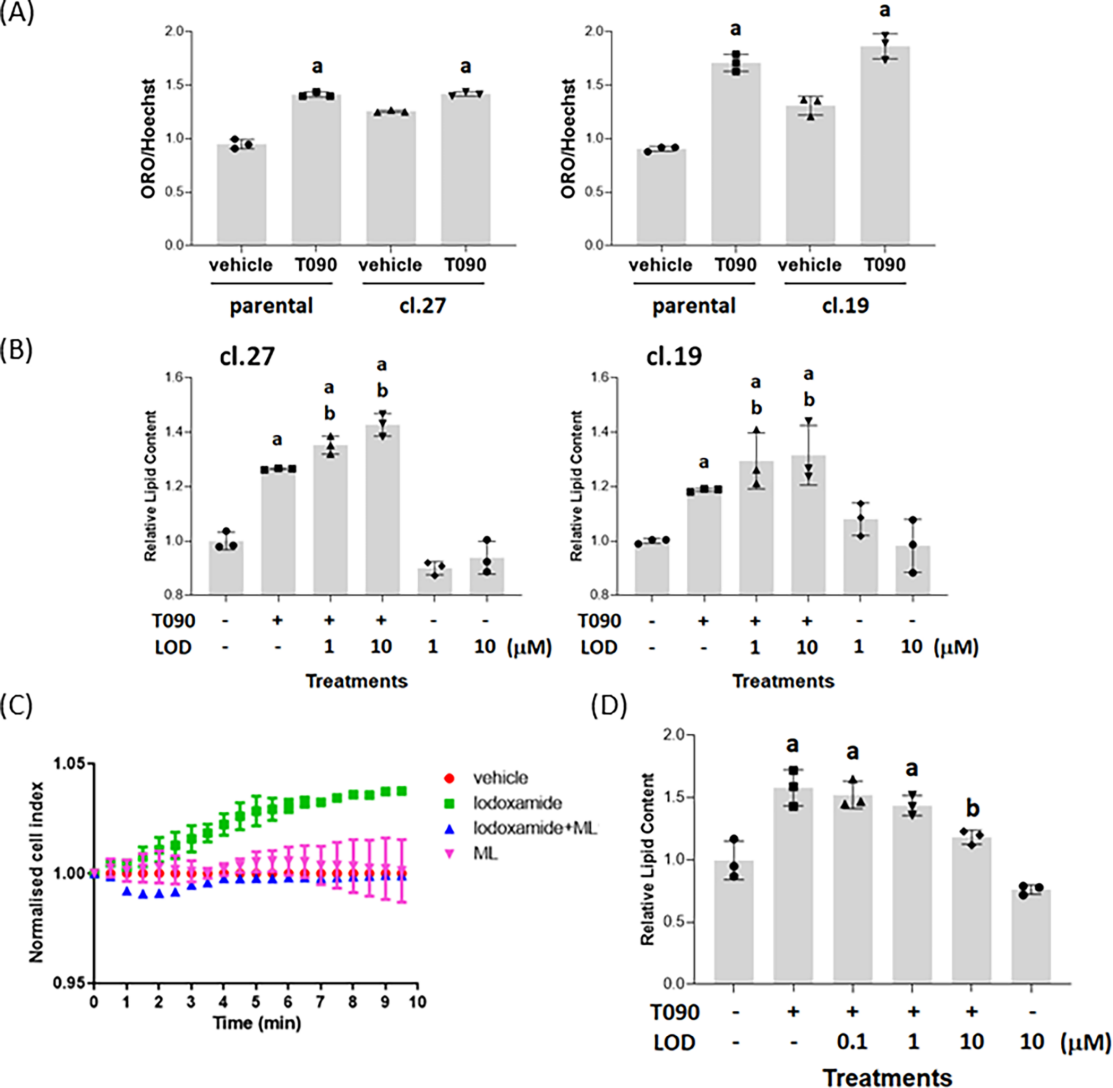
Lodoxamide regulation of the lipid level in HepG2 clones requires GPR35. Relative Oil Red O staining per cell was measured in each of parental HepG2 cells and clones 19 and 27, both basally and in response to treatment with T0901317 (4 × 10^–6^ M, 48 h) (A). a: *p* < 0.05 versus vehicle. (B) Either clone 27 (left-hand side) or clone 19 (right-hand side) cells were exposed to T0901317 with or without lodoxamide (1 × 10^–6^ M or 1 × 10^–5^ M) or with lodoxamide alone. a: *p* < 0.05 versus vehicle, b: *p* < 0.05 versus T0901317. (C) Cells of clone 27 were transiently transfected with human GPR35a and then used to record electrical impedance over time. Lodoxamide (1 × 10^–8^ M) stimulated signal and this was blocked by co-addition of ML-145 (1 × 10^–5^ M). (D) These cells were exposed to T0901317 with or without lodoxamide and relative lipid content was assessed as in (B).

In the two separate GPR35 knock-out clones of HepG2 cells, while LXR activation with T0901317 was still able to promote lipid accumulation (although as noted above from a higher baseline, resulting in a smaller window of effect when exposed to the LXR agonist for the same period of time) (Figure 4A), co-addition of lodoxamide was now unable to suppress the effect of T0901317 (Figure 4B). Importantly, as genome-editing strategies can cause unanticipated effects on the expression of “off-target” genes^30,31^ that may also influence cellular function, we transiently reintroduced human GPR35, in this case as the shorter GPR35a splice variant because it has higher signal transduction effectiveness than GPR35b.^16^ Such introduction of GPR35a into cells of clone 27 restored the ability of lodoxamide to regulate electrical conductance (Figure 4C), while this was not observed when lodoxamide was added along with ML-145 (Figure 4C). Now lodoxamide was again able to prevent the lipid accumulation induced by exposure to T0901317 in these GPR35a-reconstituted cells (Figure 4D).

Based on these underpinning studies, which provided strong evidence of a key role for GPR35 in HepG2 cells, we wished to explore whether there might be an equivalent role of GPR35 in primary hepatocytes. Following isolation of the liver from mice, we used quantitative-RT-PCR (qRT-PCR) to confirm the expression of GPR35 in this tissue and, as a positive control, in the colon (Table 1). Moreover, hepatocytes isolated following collagenase digestion of the liver also expressed a significant level of mRNA encoding mouse GPR35 (Table 2). It is well established that in addition to the essentially complete species selectivity of the human GPR35 antagonists CID-2745687 and ML-145 over mouse GPR35,^21^ there is frequently also marked variation in potency of agonist ligands at these species orthologues.^11^ For example, while lodoxamide is a high-potency agonist at human GPR35 (Table 3), it is some 450-fold less potent at mouse GPR35 (Table 3). Thus, to explore the potential effect of activation of GPR35 in hepatocytes taken from wild-type mice, it was necessary to identify a ligand with higher potency at this orthologue. Screening of a variety of ligands with potency at human GPR35 indicated that although some 10-fold less potent than at human GPR35, 6-butyl-4,10-dioxo-1,7-dihydro-1,7-phenanthroline-2,8-dicarboxylic acid (bufrolin) (Figure S1) indeed displayed relatively high potency at mouse GPR35 (Table 3) and that bufrolin was more than 10-fold more potent than lodoxamide at mouse GPR35 (Table 3).

**Table 1:** Species Orthologues of GPR35 are Expressed in Both the Colon and Liver of Wild-Type and hGPR35a-HA Transgenic Mice^a^

WT mice	mGPR35	actin
colon	26.13 ± 1.17	16.77 ± 1.43
liver	32.59 ± 0.88	19.36 ± 0.74

hGPR35a mice	hGPR35a	actin
colon	25.68 ± 0.61	19.81 ± 0.56
liver	33.68 ± 0.15	19.74 ± 0.52

a GPR35 mRNA levels were assessed via qRT-PCR in the colon and liver from either wild-type (WT) mice or homozygous hGPR35a-HA transgenic knock-in mice. Actin provided a house-keeping gene control. Data as cycle number are presented as mean ± SEM, *n* = 3.

**Table 2:** Mouse and Human GPR35 Primers Amplify Only the Appropriate Sequences and Confirm Expression of the Corresponding Sequence in Hepatocytes from Wild-Type and hGPR35-HA Mice^a^

primers	WT	WT	h35	h35	KO	KO
mouse GPR35	30.2	30.8	36.8	nd	nd	nd
	30.4	31.5	36.3	38.0	nd	nd
	30.1	30.7	nd	nd	37.5	39.0
human GPR35a-HA	nd	38.4	35.6	33.5	37.4	37.0
	nd	38.3	35.5	33.3	37.7	37.0
	nd	38.8	35.2	33.0	36.8	37.0
actin	15.2	16.8	16.2	15.5	18.2	16.1
	15.3	17.0	14.9	16.0	18.3	15.6
	15.2	16.7	16.1	15.2	17.7	15.8

a Primers designed to selectively amplify cDNA corresponding to mGPR35 and hGPR35a-HA were used on samples prepared from hepatocytes isolated from wild-type (WT), hGPR35a-HA (h35), or GPR35 knock-out (KO) mice. Data from three technical replicates of two separate hepatocyte preparations are shown. Data are cycle numbers. nd = not detected. Actin was used as a house-keeping control gene.

**Table 3:** Comparison of Ligands With Agonist Potency at Human and Mouse GPR35

	human GPR35a, pEC_50_	mouse GPR35, pEC_50_	ratio (log) (H/M)
lodoxamide	8.38 ± 0.02	5.72 ± 0.03	2.66 ± 0.05
bufrolin	7.83 ± 0.02	6.81 ± 0.04	1.02 ± 0.06
zaprinast	5.59 ± 0.01	6.18 ± 0.01	–0.59 ± 0.0

**Figure 5:**
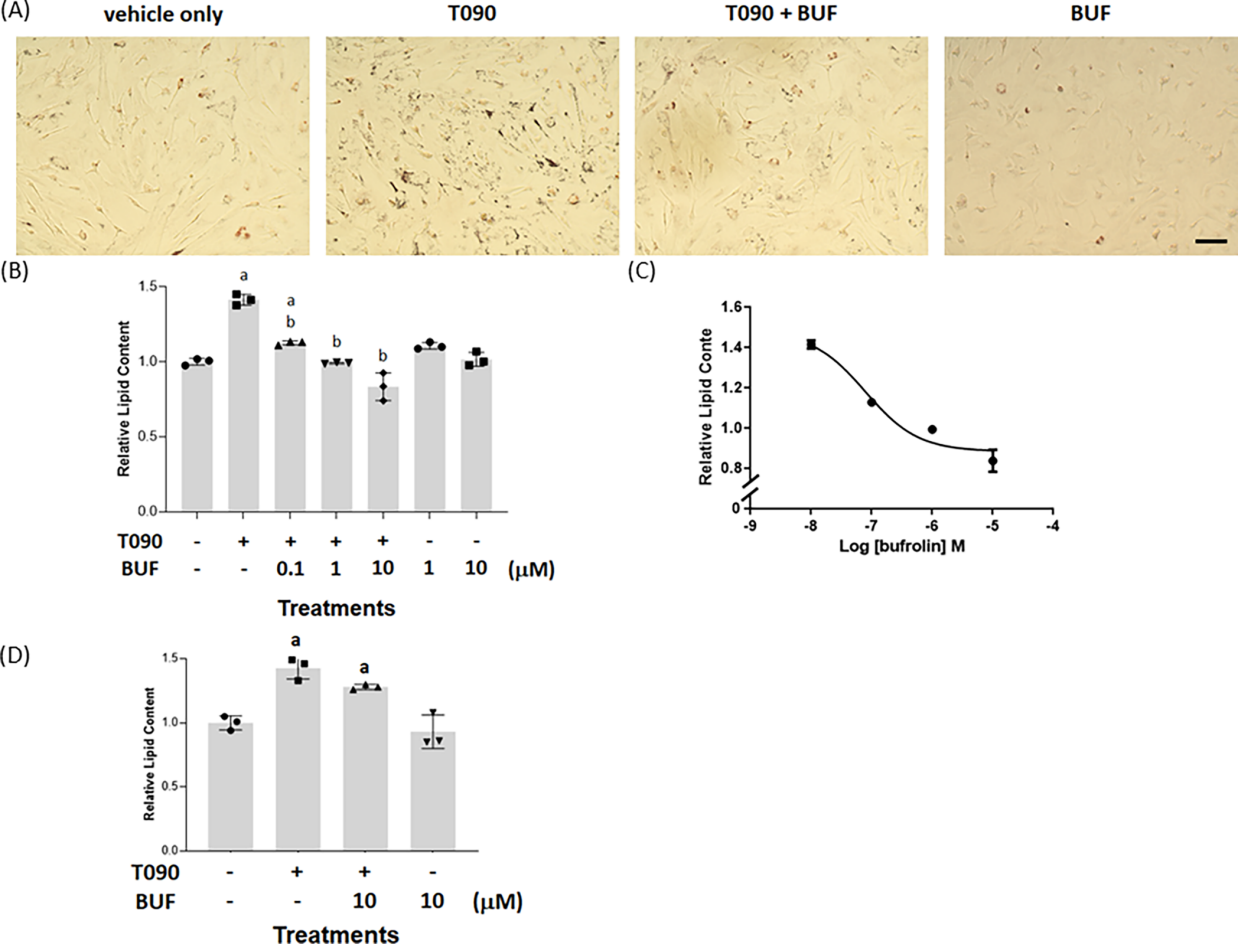
Bufrolin suppresses LXR-mediated lipid accumulation in hepatocytes from wild-type mice. Hepatocytes (scale bar = 100 μm) from wild-type mice were exposed to T0901317, T0901317/bufrolin, or bufrolin alone. Lipid content was then measured. (A) Representative images visualized (BUF = bufrolin = 1 × 10^–5^ M). (B) Oil Red O was solubilized and quantified by measuring absorbance at 510 nm, *p* < 0.05 a: versus vehicle, b: versus T0901317. The effect of varying concentrations of bufrolin (EC_50_ = 7.9 ± 0.06 × 10^–8^ M) is shown (C). Data are mean ± SEM, *n* = 3 (B, C). (D) Hepatocytes from GPR35 knock-out mice were treated as in (B) with T0901317, T0901317/bufrolin, or bufrolin alone, except that treatment with the ligands was for 5 days because a significant effect of T0901317 was not observed by treatment for 48 h. a: *p* < 0.05 versus vehicle. No significant difference (*p* > 0.05) was recorded between hepatocytes treated with T0901317 and T0901317/bufrolin.

Addition of T0901317 (5 × 10^–6^ M, 48 h) to mouse hepatocytes maintained in culture was, like in HepG2 cells, able to promote lipid accumulation in these cells (Figure 5A,B). Co-incubation of hepatocytes with combinations of T0901317 and varying concentrations of bufrolin resulted in a concentration-dependent suppression of LXR-mediated lipid accumulation (Figure 5B,C) with potency (EC_50_ = 7.87 ± 0.06 × 10^–8^ M) (Figure 5C) in line with that measured *in vitro* at mouse GPR35 (Table 3). However, because neither CID-2745687 nor ML-145 act as effective antagonists at the mouse GPR35,^21,23^ we were unable to use these compounds to ascertain with clarity whether this effect of bufrolin truly reflected an “on-target” effect at GPR35 or an undefined “off-target” effect of the compound.

We attempted to resolve this question by isolating hepatocytes from GPR35 knock-out mice. As anticipated, we were unable to detect mRNA encoding GPR35 in hepatocytes from these animals (Table 2). Bufrolin did not suppress lipid accumulation induced by T0901317 in such cells (Figure 5D), but with an equivalent time of exposure to the LXR activator as in hepatocytes from wild-type mice, the effect induced by T0901317 in hepatocytes from GPR35 knock-out mice was modest. The basis for this was not explored in detail but we note that ref (32) has reported that basal triglyceride levels are also higher in the liver of GPR35 knock-out animals than wild-type mice, and we had noted earlier that basal lipid content was higher in both the GPR35 knock-out clones of HepG2 cells than in the parental HepG2 cells (Figure 3C).

**Figure 6:**
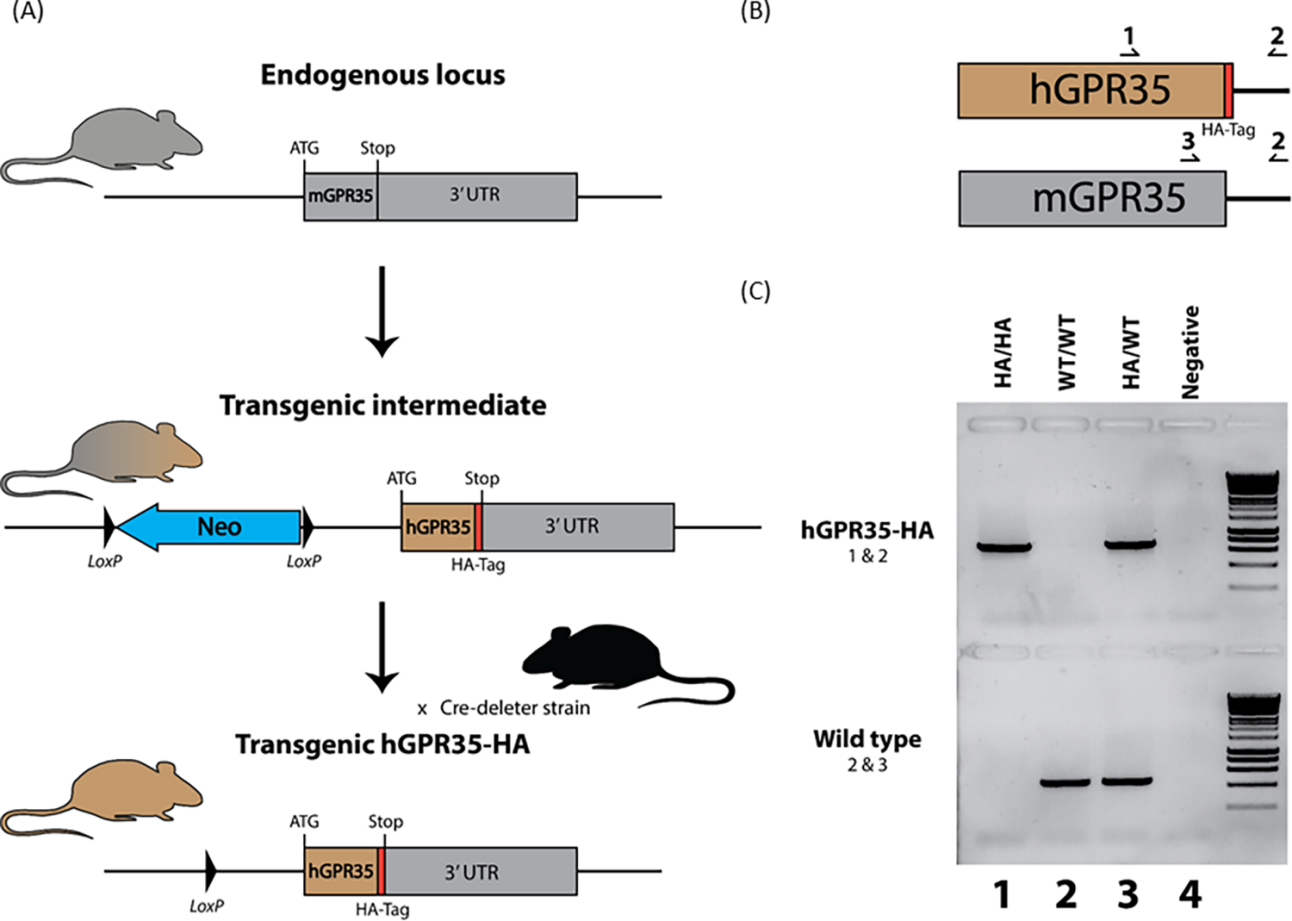
Generation and characterization of human GPR35a-HA expressing transgenic knock-in mice. (A) Transgenic C57BL/6N mice with the human GPR35a coding sequence replacing the mouse sequence, along with the addition of a HA epitope tag before the stop codon were generated via a neomycin selective gene containing intermediate. These mice were crossed with a cre-deleter line to excise the selective gene leaving only LoxP sequences and the desired hGPR35a-HA sequence. The cre-deleter was then backcrossed out to isolate the hGPR35a-HA transgenic mice. (B, C) Human- and mouse-specific forward primers were used along with a reverse primer located in the 3′ UTR to genotype transgenic homozygous (HA/HA), transgenic heterozygous (HA/WT), and wild-type (WT/WT) mice. Expression of GPR35 in humanized transgenic mice as well as wild-type mice was assessed using qRT-PCR (see Tables 1 and 2).

To allow detailed pharmacological examination of a true contribution of GPR35 to the regulation of the liver lipid content in mouse hepatocytes, we thus generated a transgenic “knock-in” line of mice in which the coding sequence of mouse GPR35 was replaced by a sequence able to encode the human GPR35a splice variant (Figure 6). Within this, we also added an in-frame HA-epitope tag sequence to the receptor intracellular C-terminal tail, as we have done with other GPCRs knocked-in to the appropriate genomic locus in mice.^33^ Following genotyping and the identification of mice homozygous for expression of human GPR35a-HA, we then examined the profile of human GPR35a-HA mRNA across tissues and compared this with the expression of mouse GPR35 in tissues from wild-type animals. We first confirmed that the primers employed for qRT-PCR to detect mGPR35 in wild-type animals were unable to amplify cDNA generated from the colon of GPR35a-HA homozygous mice, and that the reverse was also the case, i.e., that primers selected to amplify cDNA produced from the colon of hGPR35a-HA-expressing animals did not detect mGPR35 (Table 2). In hepatocytes from hGPR35a-HA, homozygous mice mRNA corresponding to this protein was detected effectively by the human orthologue specific primers (Table 2). Further tissue analysis showed an equivalent tissue expression pattern of the two GPR35 orthologues between wild-type and the hGPR35a-HA transgenic animals (not shown).

**Figure 7:**
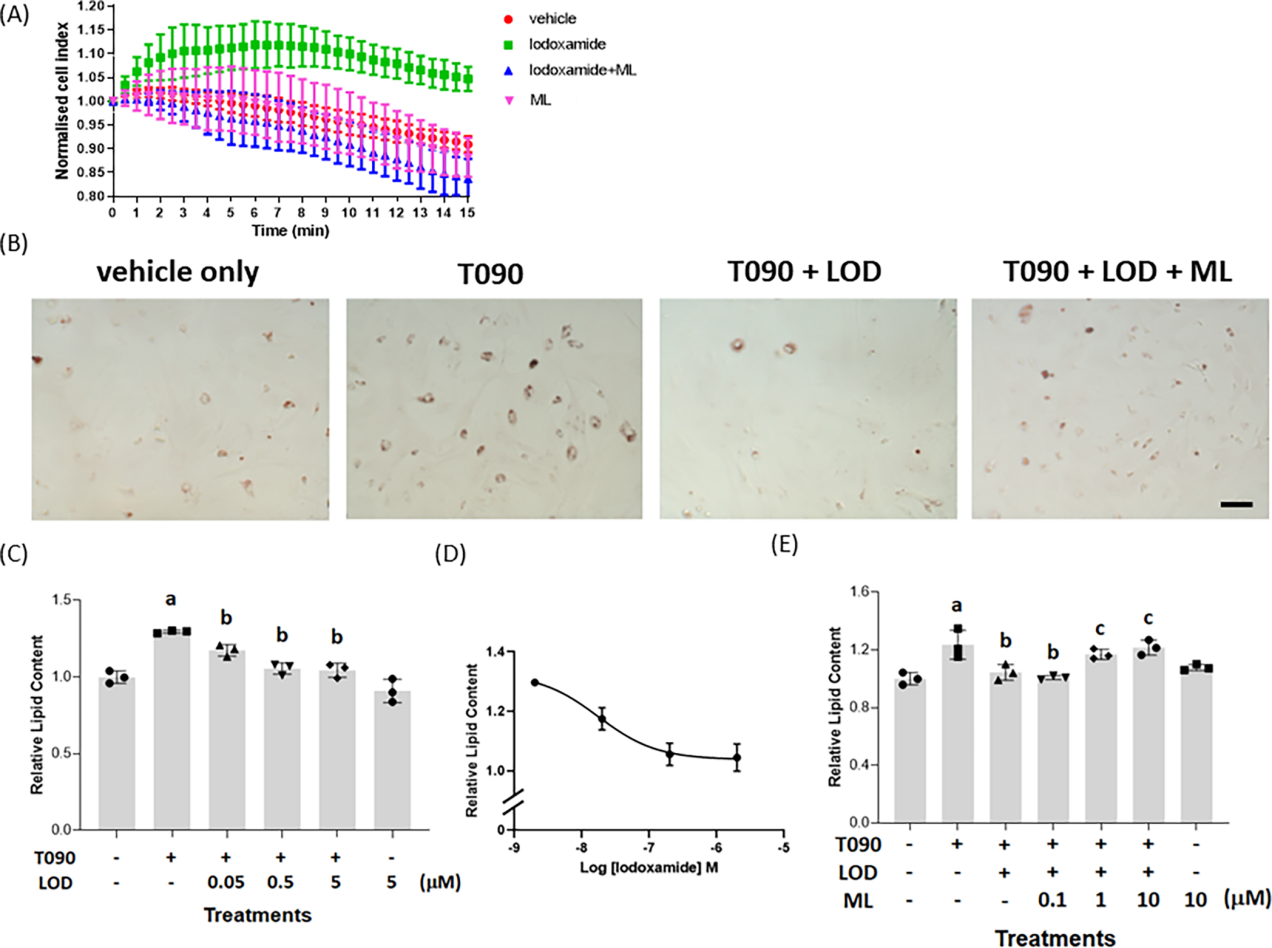
GPR35 activation suppresses LXR-induced lipid accumulation in hepatocytes from human GPR35a-HA expressing mice. Hepatocytes from GPR35a-HA expressing transgenic knock-in mice were used to assess changes in electrical impedance in response to lodoxamide (1 × 10^–7^ M) (A). Such signal was absent with co-addition of ML-145 (1 × 10^–5^M) (ML) (A). Hepatocytes (scale bar = 100 μm) isolated from these animals were maintained in culture and exposed to vehicle, T0901317 (8 × 10^–6^ M) (T090), T0901317/lodoxamide (5 × 10^–6^ M) (LOD), or T0901317/lodoxamide/ML-145 (1 × 10^–5^ M). (B) Representative visual images. Data quantified as relative lipid content (C). *p* < 0.05, a: versus vehicle, b: versus T0901317. (D) Effect of varying concentrations of lodoxamide is shown (EC_50_ = 1.7 ± 0.03 × 10^–8^ M). (E) ML-145 blocked the effect of lodoxamide in a concentration-dependent manner. *p* < 0.05, a: versus vehicle, b: versus T0901317, and c: versus lodoxamide.

With confidence in an appropriate expression pattern for human GPR35a-HA in these transgenic animals, we then generated hepatocytes from the hGPR35a-HA-expressing mice. These were used initially in label-free electrical conductance assays. Lodoxamide (1 × 10^–7^ M) generated a time-dependent increase in cellular impedance that was similar in characteristics to the response pattern recorded in HepG2 cells (Figure 7A). Moreover, as in parental HepG2 cells, this effect was prevented by the co-addition of ML-145 (Figure 7A). Once again ML-145 produced no separate effect that was distinct from addition of only the DMSO-containing vehicle solution (Figure 7A). Addition of T0901317 to hepatocytes taken from hGPR35a-HA-expressing mice resulted in a clear increase in lipid accumulation as measured by staining with Oil Red O both visually (Figure 7B) and more quantitatively following extraction from the cells (Figure 7C). This effect of T0901317 was prevented by the co-addition of lodoxamide (Figure 7B,C) and the concentration dependence of lodoxamide (EC_50_ = 1.7 ± 0.03 × 10^–8^ M) (Figure 7D) was consistent with the observed potency of this ligand at human GPR35a. Further confirmation that this reflected a GPR35-mediated effect of lodoxamide in hepatocytes derived from hGPR35a-HA-expressing mice was that the effect of lodoxamide was prevented by the additional presence of ML-145 (Figure 7B,E), which once more was without effect when added alone (Figure 7E).

**Figure 8:**
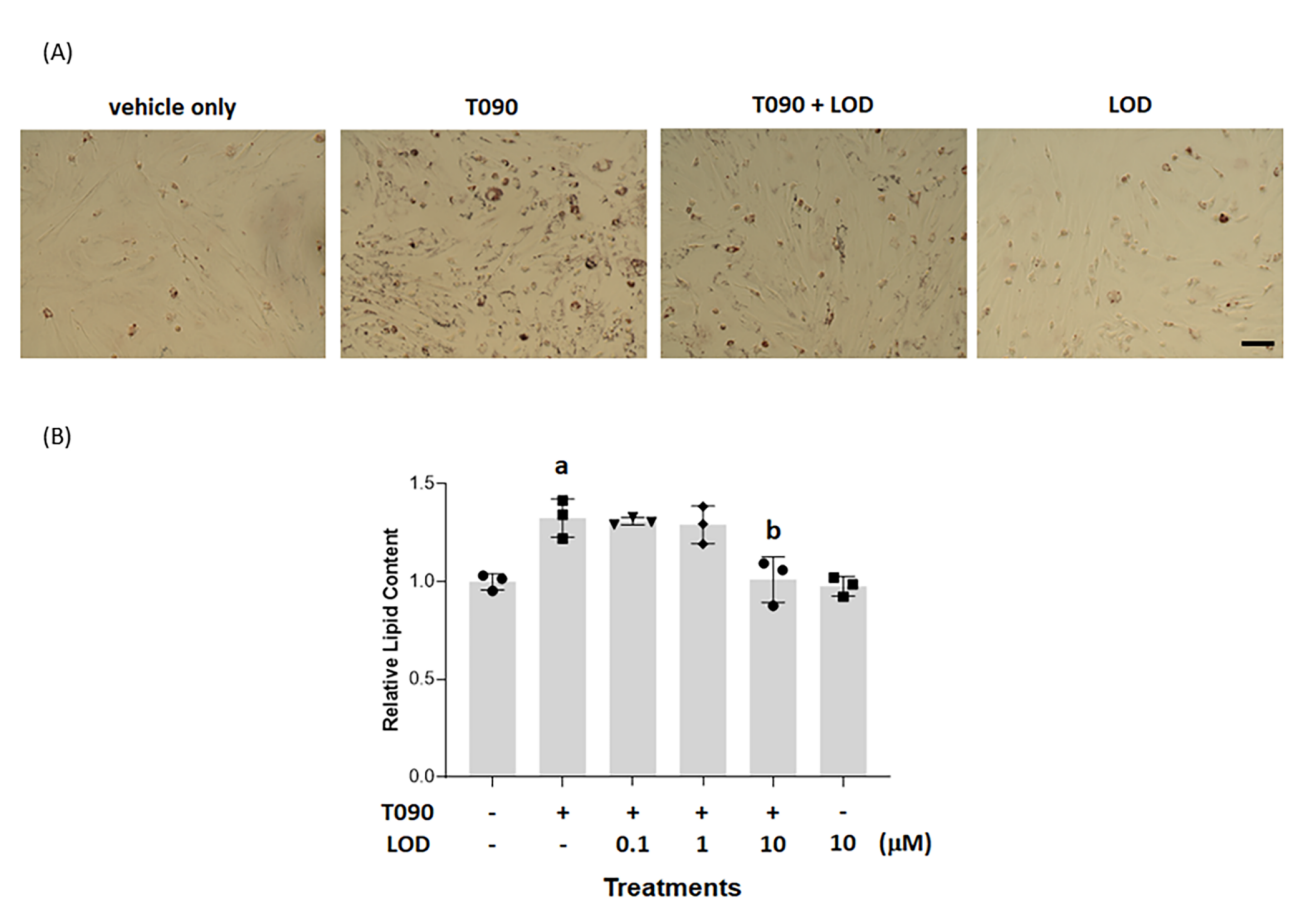
Treatment with lodoxamide can reverse pre-established lipid accumulation in hepatocytes. Hepatocytes from GPR35a-HA-expressing transgenic knock-in mice (scale bar = 100 μm) were maintained in culture and exposed to medium (vehicle only) or T0901317 (8 × 10^–6^ M, 48 h) (T090). Lodoxamide (1 × 10^–5^ M) (LOD) in (A), concentrations as indicated in (B), was then added with or without continued exposure to T0901317. (A) Representative visual images. Data quantified as relative lipid content (B), *p* < 0.05 a: versus vehicle, b: versus T0901317.

In each of the studies detailed above, the effects of potential GPR35 ligands were assessed when co-added with the LXR activator. However, in any disease treatment setting, medicines would be delivered after diagnosis rather than prophylactically. To assess if activation of GPR35 could reverse pre-established lipid accumulation, we treated hepatocytes isolated from hGPR35a-HA-expressing mice with T0901317 for 2 days to induce lipid accumulation and then added lodoxamide, in addition to T0901317, to mimic treatment in the presence of ongoing LXR activation. This too was sufficient to reduce lipid levels to those observed in the absence of T0901317 (Figure 8A) and the effect of lodoxamide was once again concentration-dependent (Figure 8B). In this case, however, lodoxamide was less potent than when added alongside T0901317 at the induction of lipid accumulation and this may indicate that greater receptor occupancy of GPR35 is required in this experimental setting.

## Discussion

The development of both NAFLD and nonalcoholic steatohepatitis (NASH), in which accumulation of triglycerides and other fats in the liver is exacerbated by inflammation and fibrosis, is attracting great interest as a disease area, which may have the potential to be treated with medicines that impact metabolic effects associated with type II diabetes.^34−36^ Alongside such studies, there is considerable interest in other pharmacological avenues to reduce lipid accumulation in hepatocytes and simple hepatocyte-like cell models can provide a useful starting point for such studies. Recently, ref (37) suggested that activation of the orphan receptor GPR35 in both human Hep3B cells and primary mouse hepatocytes could limit lipid accumulation induced by stimulation of nuclear LXR with the synthetic agonist T0901317. While the observations in human Hep3B cells were consistent with an important role for GPR35, the results in mouse hepatocytes were more challenging to interpret because human and mouse orthologues of this receptor have very distinct pharmacological characteristics.^10,11^ Not least, these include that the ligand CID-2745687, although a high-affinity antagonist of human GPR35,^20,21,24^ lacks substantial affinity for mouse GPR35.^21,23^ In the studies of Nam et al.,^37^ CID-2745687 was reported to block with high affinity the effect of a GPR35 agonist in primary mouse hepatocytes. As this is incompatible with the known pharmacology of CID-2745687, we thus determined to define conclusively if GPR35 is indeed a suitable target via which to limit lipid accumulation in hepatocytes. Initially, we employed each of wild-type human HepG2 cells and clones of these cells, which we genome-edited to eliminate expression of full-length functional GPR35. We then moved to studies of hepatocytes, initially taken from either wild-type or GPR35 knock-out mouse lines. Finally, to support key pharmacological conclusions, we generated a transgenic knock-in line of mice in which we replaced mouse GPR35 with the sequence able to encode human GPR35a, the splice variant most similar to the mouse receptor orthologue.^11,16^ Studies on hepatocytes from these humanized transgenic mice were fully supportive of a key role of GPR35 in limiting lipid accumulation, while this could not be defined clearly in studies using the tissue from wild-type mice.

An initial difference in the current studies from those of ref (37) was although they reported the expression of both the long, GPR35b, and the shorter GPR35a human splice variants of GPR35 in both HepG2 (and Hep3B) cells when using HepG2 cells of varying passage number we were only able to detect expression via PCR of the longer, GPR35b, variant. This was not an issue based on primer design as we were able to record the selective expression of GPR35a by the monocyte-like cell line THP-1. This potential difference should, however, not be anticipated to have functional consequences for comparison between the studies because the two human splice variants have equivalent pharmacology, activate the same G proteins, and interact with arrestins in a similar fashion.^16^ The only significant difference noted to date is that the signaling effectiveness of GPR35a is substantially greater than that of GPR35b.^16^

Our studies conducted with both wild-type HepG2 cells and a pair of clones generated from cells genome-edited to eliminate full-length GPR35 confirmed the lack of ability of an agonist with high potency at human GPR35 to function in the genome-edited clones. However, this was restored following transient (re)introduction of human GPR35a into these cells. As noted earlier, lodoxamide, which, as alomide, is used clinically to treat allergic keratoconjunctivitis,^38^ is a high potency activator of human GPR35. It also has similar and high potency at the rat orthologue.^15^ This, however, is not true at the mouse orthologue (Table 3). It is worthy of note that although we used β-arrestin-based interaction assays to define the potency of both lodoxamide and bufrolin at human and mouse GPR35 in these studies we have previously shown that the EC_50_ values reported herein are entirely in accord with those for these two ligands at the mouse and human GPR35 in assays that report G protein activation.^23^ We can, therefore, exclude any potential of “ligand bias”^39−41^ in providing inconsistent pharmacology at the receptor. In these various assays, lodoxamide shows some 500-fold lower potency at the mouse than at human GPR35. It is hence very poorly suited to be used for studies in mouse-derived cells and tissues. Nam et al.^37^ reported EC_50_ as 6.1 × 10^–9^ M for lodoxamide to limit lipid accumulation in mouse hepatocytes. This is simply not consistent with significant occupancy of the mouse receptor (Table 3). Moreover, they reported an IC_50_ value for CID-2745687 of 9.8 × 10^–8^ M to block mouse GPR35 (against 1 × 10^–6^ M lodoxamide) in primary hepatocytes. Once again this is not compatible with published pharmacological details of this receptor, which show this ligand to have no significant affinity at the mouse GPR35.^21,23^

To assess and potentially overcome this issue, we examined a number of other ligands with agonist potency at GPR35 to attempt to identify a high-potency agonist at the mouse orthologue. This was not particularly successful. The most potent ligand we identified in these studies was bufrolin. However, even bufrolin displayed EC_50_ < 1 × 10^–7^ M at the mouse GPR35 and it was indeed some 10-fold more potent at the human receptor. This required us to use high concentrations of bufrolin to potentially obtain substantial receptor occupancy. We did perform a group of experiments on isolated mouse hepatocytes using bufrolin. Here, by using high concentrations of bufrolin we observed a suppression of lipid accumulation with EC_50_ close to 8 × 10^–8^ M. While consistent with a role of GPR35, we considered that a lack of a suitable antagonist to attempt to block the effect of bufrolin was too limiting to take this further, despite bufrolin lacking a statistically significant effect in hepatocytes from GPR35 knock-out mice.

To extend the studies in a physiological context, we then generated a transgenic knock-in line of mice in which we replaced mouse GPR35 with the human GPR35a splice variant. Detailed expression analysis in homozygous animals showed expression of the transgene in place of mouse GPR35 in appropriate tissues and to similar levels. We anticipated that if effects on hepatocytes from the knock-in line were indeed to be mediated by the transgene, they should display appropriate human GPR35 compatible pharmacology. This was indeed the case. Initially, label-free measures of electrical conductance showed effects of low concentrations of lodoxamide that were blocked by the presence of the human GPR35 antagonist ML-145, consistent with activation of GPR35 in these cells. Equally important, lodoxamide-mediated suppression of LXR-induced lipid accumulation was concentration-dependent and occurred with EC_50_ = 1.7 × 10^–8^ M, in line with the potency of lodoxamide at human GPR35a (Table 3), and this effect was blocked with increasing concentrations of ML-145.

To extend the studies further and to assess whether treatment with a suitable GPR35 agonist could reverse preinduced lipid accumulation, as might be expected to be required for the treatment of pre-existing disease, we induced LXR activation in isolated human GPR35a-expressing hepatocytes for 48 h and following the demonstration that lipid levels had indeed increased, we then provided lodoxamide alongside the continued presence of the LXR activator. Within a 24–48 h period, lipid levels were reduced to basal, suggesting that GPR35 treatment could potentially reverse disease rather than simply acting as a prophylactic treatment. Finally, it is of great interest that ref (42) reported beneficial effects of the treatment of high-fat diet-induced wild-type mice with oral lodoxamide at 1 mg/kg for the final 7 days of a 7-week-diet exposure treatment. Although lodoxamide is used as a topically applied medicine, we are unaware of any pharmacodynamic or pharmacokinetic data for this compound in the mouse and, therefore, it is impossible to consider whether appropriate target engagement was achieved in the studies reported. However, simple calculations hint that it is unlikely. However, with appropriate studies to determine effective dosing regimens, based on the current studies, it is likely that work to further promote the investigation of targeting GPR35 in NAFLD and/or NASH would be fruitful.

## Methods

### Chemicals and Reagents

Lodoxamide and T0901317 were purchased from Cayman Chemical (Ann Arbor, MI). Zaprinast, CID-2745687, ML-145, Pertussis toxin, and Y27632 were obtained from Tocris Bioscience (Abingdon, U.K.). Bufrolin was synthesized in collaboration with Novartis, Horsham, U.K. Oil Red O and Hoechst 33342 were from Sigma-Aldrich (St. Louis, MO). LipofectAMINE 3000 reagent was purchased from Invitrogen (Carlsbad, CA). FR900359^26^ was a gift from Dr. Evi Kostenis, University of Bonn, Germany.

### Cell Culture and Treatment

Human hepatocellular carcinoma HepG2 cells were grown in Minimum Essential Medium (MEM) supplemented with 10% fetal bovine serum (FBS), 2 mM l-glutamine, 1× non-essential amino acid, 1 mM sodium pyruvate, and 1× penicillin/streptomycin. Materials for cell culture were all from Sigma-Aldrich (St. Louis, MO) or Thermo Fisher Scientific (Waltham, MA). Cells were incubated in a humidified CO_2_ incubator at 37 °C. Experiments were performed when cell confluence reached 80%. Cells were seeded onto a 12-well plate (1 × 10^5^ cells/well) for 24 h prior to drug treatment. The cells were then incubated with T0901317 and/or GPR35 agonists/antagonists (in MEM containing 5% FBS), as indicated for 48 h.

### Generation of Genome-Edited HepG2 GPR35 KO Cells

Expression of full-length mRNA from the GPR35 gene was eliminated using a dual synthetic gRNA/RNP approach^43^ with one guide cutting the intron and one in the exon region to generate a nonfunctional GPR35 protein. The gRNAs were designed with the CRISPR Finder (Welcome Sanger Institute). The sequence of each gRNA in the GPR35 gene is provided in Table S1. HepG2 cells were transfected with the Neon Transfection System from Thermo Fisher Scientific (Waltham, MA), according to the manufacturer’s instructions. Briefly, CrRNA and tracrRNA were annealed to gRNA at 95 °C for 5 min. Next, cells were transfected with gRNA, Cas9, and the electroporation enhancer, according to the manufacturer’s instructions. All crRNAs, tracrRNA, SpCas9 nuclease, and the electroporation enhancer were purchased from Integrated DNA Technologies (Coralville, IA). Transfected cells were plated and analyzed for editing efficiency 24–48 h after electroporation. Single-cell clones were generated by single-cell FACS sorting. Clones were expanded and to access editing efficiency, genomic DNA from cell clones was extracted and subjected to PCR to confirm the genome deletion. The sequence of the PCR primers and the expected deletion sizes are listed in Table S1. To further quantify and capture the genomic DNA deletion, PCR primers listed in Table S1 were designed to amplify the genomic DNA surrounding the target site. The PCR primers were linked to the sequences of the Illumina Nextera adapters. The amplified PCR products were subjected to paired-end sequencing using NextSeq500 from Illumina using paired-end chemistry with a 150-bp read length. In addition, mRNA from KO clones was isolated and subjected to RT-PCR. The sequence of the PCR primers is listed in Table S1. To further confirm the abrogation of GPR35 activation in CRISPR KO clones, KO cells were seeded onto xCELLigence E plates (2 × 10^4^ cells/well) for 24 h at 37 °C prior to the treatment of GPR35 agonists. The impedance was recorded using xCELLigence RTCA. Each experiment was repeated at least three times.

### Reintroduction of Human GPR35a Into Genome-Edited HepG2 GPR35 KO Cells

Briefly, CRISPR KO cells were transiently transfected 24 h with the human GPR35a expression vector using LipofectAMINE 3000 reagent, according to the manufacturer’s instructions. Lodoxamide-induced human GPR35 activation in transfected cells was confirmed using the xCELLigence RTCA analysis. Alternatively, after 24 h transfection, cells were seeded onto a 12-well plate (1 × 10^5^ cells/well) for 24 h at 37 °C. Then, cells were incubated with T0901317 and/or GPR35 agonists (in MEM containing 5% FBS) for 72 h. After the drug treatment, cells were stained with Oil Red O to examine the levels of lipid accumulation. Each experiment was repeated at least three times.

### Generation of Transgenic Mice

Transgenic humanized HA-tagged GPR35 knock-in C57BL6/N mice were generated by Genoway (Lyon, France). Briefly, embryonic stem cells from C57BL6/N mice were transfected with a target cassette construct containing ∼5.8 kbp of the mouse genomic GPR35 coding region with the mouse GPR35 CDS replaced with the human GPR35a CDS with the addition of a HA tag fused C-terminally before the stop codon. This also included a *neomycin* selective gene between two LoxP sites 430bp upstream of the ATG start codon. This includes a short-arm homologous region upstream of the LoxP site (∼2.2 kb) and a long-arm homologous region downstream of the HA site (∼2.3 kb). Neomycin positive clones were screened by PCR, and confirmed cells were introduced into wild-type C57BL6/N blastocysts to produce chimera mice. These were crossed with a C57BL6/N cre-deleter strain to excise the neomycin gene before backcrossing to wild-type to produce the heterozygous transgenic mice. The genomic region was then sequenced to confirm correct knock-in and cre-excision.

GPR35 KO mice are described in ref (44).

### Genotyping of HA-Tagged Humanized GPR35 Transgenic Mice

The genomic DNA was isolated from tail tips and used to detect the humanized GPR35 transgene by PCR using a human-specific forward primer and a mouse reverse primer binding in the 3′ UTR to give a band size of 739 bp. The wild-type allele was detected using a mouse-specific forward primer and the same mouse 3′ UTR reverse primer as above to give a size of 480 bp. The sequence of the PCR primers is listed in Table S2.

### Real-Time Reverse-Transcription Polymerase Chain Reaction (qRT-PCR)

To examine GPR35 gene expression levels, total mRNA from the tissues of WT, humanized GPR35, or KO mice was converted into cDNA, as described above. qRT-PCR analysis was carried out with 65 ng of the cDNA sample using Fast SYBR Green Master Mix (Thermo Scientific) in accordance with the manufacturer’s instructions. Human or mouse GPR35-specific primers are listed in Table S1.

### Reverse-Transcription Polymerase Chain Reaction (RT-PCR)

THP-1 monocytes (express GPR35a isoform) were maintained in RPMI-1640 (Invitrogen, Waltham, MA) supplemented with 10% FBS, 2 mM l-glutamine, and 1% penicillin/streptomycin. HT-29 cells (express GPR35b isoform) were maintained in Dulbecco’s Modified Eagle Medium supplemented with 10% FBS and 1% penicillin/streptomycin. Total mRNA was isolated from the cells using the RNeasy mini kit from QIAGEN (Germantown, MD). RNA concentrations were determined by a Nanodrop ND-1000 spectrophotometer. One microgram of RNA was transcribed using the Qiagen QuantiTect Rev. Transcription Kit. Synthesized cDNA products and primers for each gene were subjected to PCR with Promega Go-Taq DNA polymerase (Madision, WI). Specific primers are listed in Table S1.

### Mouse Primary Hepatocyte Isolation and Treatment

All mice were bred as WT or homozygous onto a C57BL/6N background. Animals were cared for in accordance with national guidelines on animal experimentation. All animal experiments were conducted under a home office license held by the authors. WT, humanized GPR35a, or KO male mice at 3–4 months of age were used in this study. These mice were fed ad libitum with a standard mouse chow diet. Briefly, mouse primary hepatocytes were isolated using a two-step collagenase perfusion technique.^45^ Twelve-well plates or xCELLigence E plates were coated with rat tail collagen I solution (Thermo Fisher Scientific) overnight at 37 °C and washed with PBS. Isolated primary hepatocytes were seeded onto 12-well plates (8 × 10^4^ cells/well) or xCELLigence E plates (2 × 10^4^ cells/well) in Wiliam’s E medium (Thermo Fisher Scientific) supplemented with 10% fetal bovine serum (FBS) and 1× penicillin/streptomycin. Cells were maintained for 7 days with a fresh medium change every other day. To confirm the activation of human GPR35, humanized GPR35 hepatocytes were treated with the GPR35 agonist and/or antagonist. Human GPR35 activation was examined using xCELLigence RTCA assay. To access the impact of GPR35 agonists on the lipid accumulation, WT and humanized GPR35 hepatocytes were incubated with T0901317 and/or GPR35 agonists/antagonists, as indicated (in DMEM medium containing 5% FBS and 1.5% BSA) for 48 h. Alternatively, GPR35 KO hepatocytes were incubated with drugs, as indicated for 5 days. In the T0901317 pretreatment model, human GPR35 hepatocytes were stimulated with T0901317 for 2 days and then further treated with lodoxamide for a further 3 days. After each drug treatment, hepatocytes were stained with Oil Red O and quantified as described. Each experiment was repeated at least three times.

### Oil Red O Staining

Lipid accumulation was measured by Oil Red O staining. A working solution of Oil Red O was prepared, as described with modification.^46^ After drug treatment, cells were washed with PBS and fixed with formalin solution for 1 h at 4 °C. Then, cells were stained with Oil Red O working solution for 10 min at room temperature. After PBS washing, Oil Red O was extracted from cells using 150 μl of isopropanol and OD_510 nm_ measured. After the lipid extraction, cells were further soaked in PBS overnight at 4 °C. These cells were then stained with Hoechst 33342 (1 μg/mL) and the fluorescence intensity of Hoechst (represents the DNA content in cells) was measured using a CLARIOstar plate reader (BMG LABTECH). Oil Red O accumulation was corrected for differences in DNA content and expressed as relative absorbency, taking the control condition (treated with vehicle only) as 1.

### Label-Free Impedance Assays

HepG2 cells were seeded onto xCELLigence E plates (2 × 10^4^ cells/well) and incubated for 24 h at 37 °C. To evaluate GPR35 activation and its downstream G protein signaling, HepG2 cells or primary mouse hepatocytes were exposed to GPR35 antagonists or G protein inhibitors 1 h (overnight for Pertussis toxin) prior to GPR35 agonist stimulation. After the addition of GPR35 agonist or vehicle control, the impedance (represented as “cell index”) was recorded every 30 s over a 2 h period using xCELLigence RTCA (Agilent). Each experiment was repeated at least three times.

### Statistical Analysis

Student’s two-tailed *t*-test was used for the determination of statistical relevance between groups, and *p* < 0.05 was considered statistically significant. All statistical analyses were performed with GraphPad Prism software.

## Supporting Information Available

Figure S1: chemical structures of GPR35 agonists, antagonists, and an LXR activator; Figure S2: CRISPR-Cas9 genome editing produces HepG2 clones lacking expression of GPR35. HepG2 cells were subjected to CRISPR-Cas9-mediated genome editing targeting the GPR35 gene. Next-generation sequencing of various clones identified disruption of the GPR35 gene sequence within the open-reading frame. (A) Clone 27 contains 1 larger deletion and 1 smaller deletion in the exon and which is out of frame (upper), while clone 19 contains 1 large deletion and 3 smaller deletions (lower) that all are within the exon and out of frame. (B) RT-PCR confirmed deletion of the sequence of GPR35b in both these clones but was able to detect a fragment of smaller size than for full-length GPR35b. hGAPDH served as a control; Table S1: primers used in the production of genome-edited clones of HepG2 cells; Table S2: primers used for expression analysis of human and mouse GPR35 (PDF)
