# Supplementary material for: G Protein-Coupled Receptor GPR35 Suppresses Lipid Accumulation in Hepatocytes
Source: ACS Pharmacol Transl Sci. 2021 Nov 30;4(6):1835–48. doi: 10.1021/acsptsci.1c00224 (PMC8669712; doi:10.1021/acsptsci.1c00224)
Supplement: Supplementary file 1 — pt1c00224_si_001.pdf [file pt1c00224_si_001.pdf]

Supporting Information

The G protein-coupled receptor GPR35 suppresses lipid accumulation in hepatocytes

Li-Chiung Lin[1], Tezz Quon[1], Susanna Engberg[2], Amanda E. Mackenzie[1], Andrew B. Tobin[1] and Graeme Milligan[1*]

1. The Centre for Translational Pharmacology, Institute of Molecular, Cellular and Systems Biology, College of Medical, Veterinary and Life Sciences, University of Glasgow, Glasgow G12 8QQ, United Kingdom
2. Discovery Biology, Discovery Sciences, R&D, AstraZenena, Gothenburg 431 50, Sweden

***Address correspondence to Graeme Milligan (Graeme.Milligan@glasgow.ac.uk)**

Table of Contents

1. Figure S1. Chemical structure of GPR35 agonists, antagonists and LXR activator (p.3)

2. Figure S2. CRISPR-Cas9 genome editing produces HepG2 clones lacking expressing of GPR35 (p.4)

3. Table S1. Primers used in the production of genome edited clones of HepG2 cells (p.5)

4. Table S2. Primers used for expression analysis of human and mouse GPR35 (p.6)

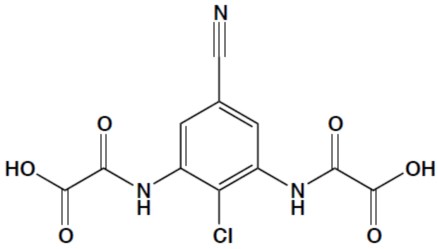

lodoxamide

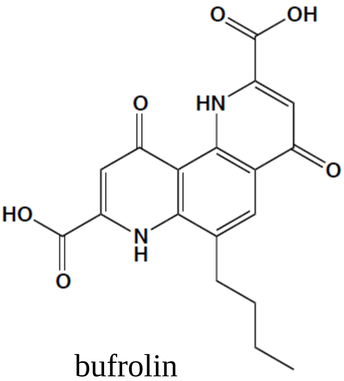

bufrolin

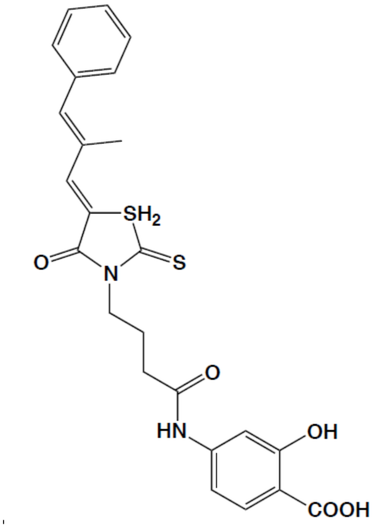

ML-145

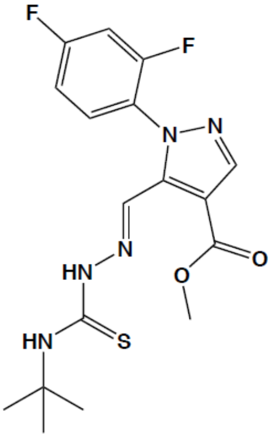

CID-2745687

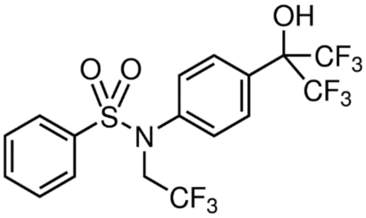

T0901317

Figure S1. Chemical structure of GPR35 agonists, antagonists and LXR activator.

(A)

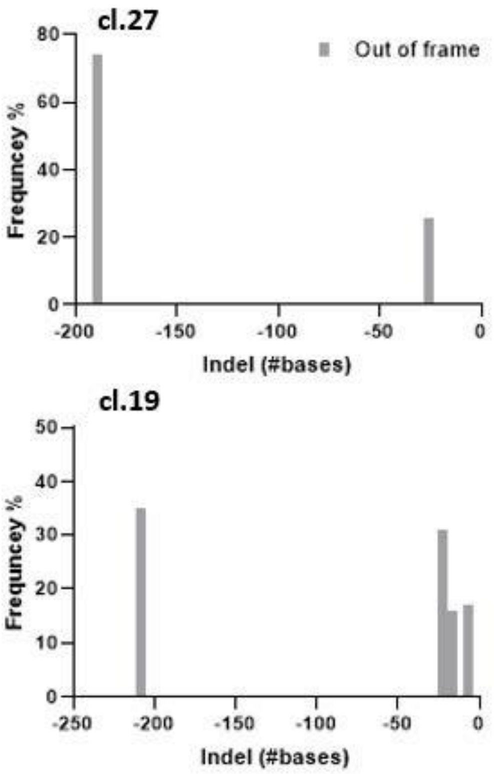

(B)

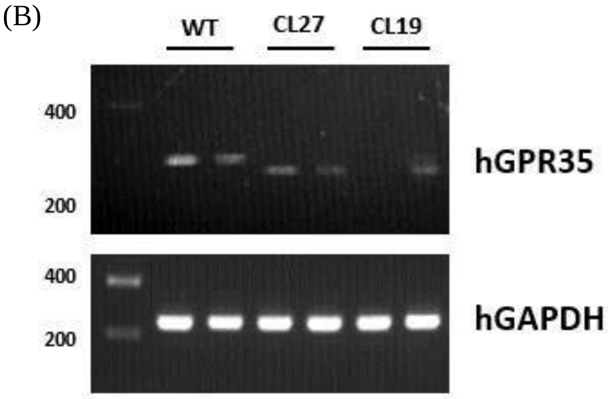

Figure S2. CRISPR-Cas9 genome editing produces HepG2 clones lacking expressing of GPR35

HepG2 cells were subjected to CRISPR-Cas9-mediated genome-editing targeting the GPR35 gene. Next generation sequencing of various clones identified disruption of the GPR35 gene sequence within the open-reading frame. **A.** clone 27 contains 1 larger deletion and 1 smaller deletion in the exon and which is out of frame (**upper**) whilst clone 19 contains 1 large deletion and 3 smaller deletions (**lower**) that all are in within the exon and out-of-frame. (**B**). RT-PCR confirmed deletion of sequence of GPR35b in both these clones but was able to detect a fragment of smaller size than for full length GPR35b. hGAPDH served as a control.

Table S1

**gRNA sequence**	
gRNA pair	AGGTCAGCAGAGAGTGAGCAGGG
	TCTCCGTCCACTGCTGCATGCGG
**PCR primers for genome deletion efficiency check**	
F'	CACCCATGCTTTCTTTGAGGAGTT (FAM labelled)
R'	AGCGGCGTGTCTGAGGTGTC
wt size: 412 bp<br>expected deletion size: 189 bp	
**NGS primers**	
F'	TCGTCGGCAGCGTCAGATGTGTATAAGAGACAGGGCTCACCTCCTCCCACATC
R'	GTCTCGTGGGCTCGGAGATGTGTATAAGAGACAGGCACAGCAGGCAGAGGTC
**RT-PCR primers**	
F'	GATCAAGCTGGGCTTCTACG
R'	CAGGCTGATGCTCATGTACC

Table S2

**qRT-PCR primers**	
human GPR35-specific	
F'	GTGCCCTCCTGGAGACGAT
R'	GCAGCAGTTGGCATCTGAGA
mouse GPR35-specific	
F'	CCAAGATTCCCAGATCCTGA
R'	CTTGCTCACATCACAGGTTCC
actin	
F'	GACAGGATGCAGAAGGAGATTACTG
R'	CTCAGGAGGAGCAATGATCTTGAT
**RT-PCR primers**	
human GPR35a	
F'	ATGCTGGCTCTTCAGAGGTG
R'	GATGACCAGCACCCAGAGG
human GPR35b	
F'	TGCTTCATAGTCCTTGCGTCTC
R'	GATGACCAGCACCCAGAGG
human GAPDH	
F'	GAGTCAACGGATTTGGTCGT
R'	TTGATTTTGGAGGGATCTCG
**Genotyping primers**	
humanised GPR35 transgene	
F'	CGGCACAATTTCAACTCCATGG
R'	GGGGAGGGGTGTATCCTAAA
wild type allele	
F'	TGAACCTCAATACCTGTGCTGC
R'	GGGGAGGGGTGTATCCTAAA
